# *Campylobacter* in an Urban Estuary: Public Health Insights from Occurrence, HeLa Cytotoxicity, and Caco-2 Attachment Cum Invasion

**DOI:** 10.1264/jsme2.ME19088

**Published:** 2019-12-27

**Authors:** Mahbubul H. Siddiqee, Rebekah Henry, Rhys A. Coleman, Ana Deletic, David T. McCarthy

**Affiliations:** 1 Environmental and Public Health Microbiology Laboratory EPHM Lab, Department of Civil Engineering, Monash University Clayton, VIC-3800 Australia; 2 Molecular and Environmental Microbiology Laboratory MEM LAB, Department of Mathematics and Natural Sciences, BRAC University Dhaka-1212 Bangladesh; 3 Melbourne Water Corporation Docklands, VIC-3008 Australia

**Keywords:** recreational water, fecal contamination, fecal pathogens, *Campylobacter* detection, gentamicin protection assay

## Abstract

Aquatic recreation in urban estuaries worldwide is often restricted by fecal pollution. Variability in the occurrence of fecal pathogens and their differential virulence potentials within these estuaries may result in variable public health risks. To address this hypothesis, *Campylobacter* were isolated from the Yarra River estuary, Australia and then characterized via HeLa cell cytotoxicity and attachment to and the invasion of Caco-2 monolayers. Overall, 54% (*n*=216) of estuarine samples (water and sediment combined) yielded biochemically confirmed culturable *Campylobacter*; higher detection was recorded in water (92%, *n*=90) than in the bank and bed sediments combined (27%, *n*=126). The seasonality of occurrence was not significant. HeLa cell cytotoxicity revealed that estuarine *Campylobacter* had low cytotoxin titers; the 95% confidence interval (CI) ranged between 61 and 85, which was markedly lower than the mean value (~386) for the *C. jejuni* 11168 reference pathogenic strain. The Caco-2 attachment of estuarine *Campylobacter* isolates (*n*=189) revealed that the 95%CI for the attachment efficiency of the test strains ranged between 0.09 and 0.1%, with only 3.7% having a higher efficiency than the 5^th^ percentile value for *C*. *jejuni* 11168. None of the estuarine strains exhibited Caco-2 invasion capabilities. In contrast to the common assumption during quantitative microbial/risk assessments (QMRAs) that all environmental strains are pathogenic, the present results revealed that *Campylobacter* within the Yarra River estuary had very low virulence potential. Since this is the first study to use human epithelial cell lines to characterize estuary-borne pathogens, these results generate valuable insights for a better understanding of the public health risks in urban estuaries that will underpin more robust QMRAs.

The fecal contamination of recreational water bodies, such as urban estuaries, is a major concern for public health. For example, fecal microbes, such as bacteria and viruses, originate from their mammal or avian hosts and, amongst other diseases, may cause severe gastrointestinal illnesses (30, 35, 79). These microbes may be introduced into natural waters via a number of different routes, including the direct deposition of fecal matter into the water body or treated effluent discharges from wastewater treatment plants or within stormwater runoff (64). During water-related recreational activities, the drinking or accidental ingestion of contaminated water with fecal organisms, including *Campylobacter*, which has a generally low infection dose (38, 50), is a common route of exposure that may result in a range of diseases (69). Infection with *Campylobacter* is one of the leading causes of gastrointestinal illness worldwide (11, 44). In Australia, campylobacteriosis comprises one of the two most frequent bacterial gastrointestinal infections (along with salmonellosis), and while the vast majority of these cases are from exposure to contaminated food or poor personal hygiene, there is increasing evidence for a link to water as a potential source (44, 58, 63). Surface water is frequently implicated in epidemiological investigations with *Campylobacter* infections (6, 11, 16).

Urban estuaries provide opportunities for multiple water-based recreational activities (*e.g*., swimming, water skiing, rowing, and fishing) in many cities throughout the world. While the findings of some studies have provided an understanding of *Campylobacter*-related human health risks in urban estuaries, they focused on the occurrence of this potential pathogen in the water column (6, 33, 40, 71, 72, 78). However, estuaries comprise multiple potential habitats for *Campylobacter* in addition to the water column, including bed sediments and intertidal bank sediments (61). These habitats differ from each other based on parameters such as moisture availability, salinity, dissolved oxygen, and chemical composition as well as exposure to atmospheric parameters; *e.g*. temperature, solar radiation, and relative humidity (15, 32). These factors may positively or negatively influence the occurrence and survival of fecal microorganisms (7, 15, 20, 67). This interplay among these physicochemical factors typically results in spatial and temporal fecal microbe variability within estuaries (42).

The small number of studies that have investigated the occurrence and survival of *Campylobacter* in sediments (65, 70) suggests that public health risk assessments in urban estuaries are potentially overlooking an important source of *Campylobacter* to the overlying water column, particularly via resuspension during high flow events or recreational activities. Furthermore, environmental isolates of *Campylobacter* have been reported to have variable virulence (10, 18, 24). While some *Campylobacter* are highly virulent and result in serious human illness, such as Guillain-Barre syndrome (GBS), only a small fraction are considered to be associated with illness (51, 74). Therefore, further efforts to clarify the potential public health risks of *Campylobacter* need to consider the intrinsic ability of estuarine *Campylobacter* isolates to be pathogenic to humans.

Interactions between *Campylobacter* and epithelial cell lines of human origin may offer valuable insights into the potential pathogenicity of strains under intestinal conditions (12, 18). Among *in vitro* assays, HeLa cell cytotoxicity assays are widely applied to test the capacity for exogenous cytotoxin production (28). Furthermore, a correlation has been reported between *Campylobacter* strains with higher efficiency to attach to or invade epithelial cells and virulence potential *in vivo* (9, 48, 57). Therefore, quantitative measures of the ability to invade and/or attach to human intestinal epithelial cells (*e.g*., Caco-2 and Hep-2) have been used to characterize the pathogenic potential of *Campylobacter* (49, 55).

The aim of the present study was to investigate spatial and temporal variabilities in the occurrence of *Campylobacter*, production of extracellular toxins, and attachment to and the invasion of human intestinal epithelial cells within an urban estuary—the Yarra River estuary, Melbourne, Australia.

The estuarine section of the Yarra River is reported to have elevated levels of fecal indicator microorganisms (33, 42), which frequently exceed those suitable for primary contact recreational activities, such as swimming. However, the estuary is well known for secondary contact water-based recreational activities, such as rowing, kayaking, and fishing (21), and fecal indictor levels are generally below secondary contact guidelines, except for during and immediately after wet weather. The present results may be used to improve quantitative microbial risk assessments (QMRAs), thereby providing a better estimate of human health risks for the users of this urban estuary and potentially other estuaries with similar catchment and climatic characteristics.

## Materials and Methods

### Site description

**Fig. 1:**
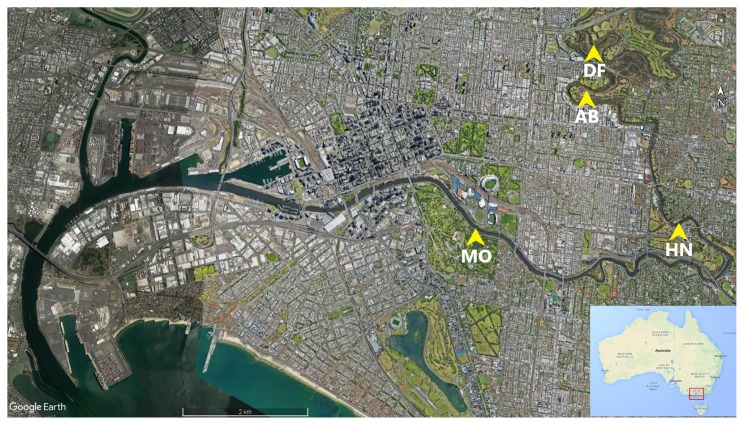
Sampling sites along the Yarra River estuary. Dights Falls (DF) marks the beginning of the estuary, and Abbotsford (AB), Hawthorn (HN), and Morell Bridge (MO) were the three estuarine sampling locations with a low to high salinity gradient, respectively (image source: Google Earth)

The Yarra River is 242 km long and flows through Melbourne, Australia with a total catchment area of approximately 4,000 km^2^. The upper reaches of this river are forested, while the middle reaches are predominantly rural before flowing through an urbanized portion within the city of Melbourne. This lower reach includes a 22-km estuary, starting just below Dights Falls (DF) and flowing into Port Phillip (Fig. 1).

Sampling locations for the present study were selected based on the salinity intrusion profile along the estuary. A calibrated 3D hydrodynamic model (41) was used to predict locations at which salinity levels were >5 ppt (to mark the intrusion of the salt wedge) at the bottom layer across the length of the estuary. Three sites that matched these criteria were selected as study sites: Morell Bridge (MB; −37.827716, 144.983926), Hawthorn (HN; −37.827419, 145.022922), and Abbotsford (AB; −37.804054, 145.001043). Dights Falls (DF; −37.7969, 145.0011) was also included to represent inputs from freshwater sources, thereby allowing for comparisons between estuary-exposed and freshwater-isolated *Campylobacter*.

### Weather conditions during sampling periods

Data for daily maximum and minimum temperatures (°C), relative humidity (%), average daily rainfall (mm), and average solar radiation (MJ m^−2^) for the days before sampling were obtained from the Bureau of Meteorology (BOM), Australia for the closest site (Melbourne, Olympic Park; station ID: 086338; http://www.bom.gov.au/climate/data-services). Furthermore, electrical conductivity (EC; mS cm^−1^), dissolved oxygen (DO; mg L^−1^), turbidity (NTU), and salinity (%) were measured using a Horiba multi-probe (HORIBA, Kyoto, Japan) during the collection of water and sediment samples.

### Collection and processing of samples

Water samples (1 L in a sterile plastic container) were collected from a depth of ~30 cm with a grab sampler, while bank and bed sediment samples (both via stainless steel corers) were aseptically collected twice a week during winter and summer from four sampling sites. The winter regime lasted from 1^st^ July 2014 to 20^th^ August 2014 and the summer regime from 1^st^ of February 2015 to 11^th^ March 2015. Fifty-one winter and 39 summer water samples were processed for the isolation of *Campylobacter*. A 300-mL subsample was filtered from each of the water samples using two nitrocellulose filters (0.45 μm, Millipore, Burlington, MA, USA). Thirty-six bank sediment and 39 bed sediment samples were collected from the three estuarine sampling locations during winter, while 25 bank sediment and 26 bed sediment samples were collected during summer for *Campylobacter* isolation. A 5-g subsample from each of the bank and bed sediment samples was homogenized in food grade plastic bags and added to a primary enrichment broth (20 mL) for *Campylobacter* isolation.

### Isolation of *Campylobacter*

The isolation of *Campylobacter* was performed according to the Australian Standard (AS) 4276.19:2001 (8), which we previously validated for our region using PCR confirmation (33). In all assays, *C. jejuni* (NCTC 11168) was used as a positive control and *Escherichia coli* (K1) as a negative control during isolation. The membrane filters for water and sediment samples were placed into 25 mL of Preston Broth and kept at 37°C for 2 h for initial resuscitation. A *Campylobacter*-specific mixture of four antibiotics (polymixin B, rifampicin, trimethoprim, and cycloheximide) (SR0117; Oxoid, Basingstoke, UK) was added to Preston broth before incubating at 42°C for 48±2 h under micro aerophilic conditions using an Oxoid CampyGen 2.5-L Sachet. After primary enrichment, 2-μL samples were subcultured (streaking method) on modified *Campylobacter* Selective Blood Free Agar (CCDA) media containing cefoperazone and amphotericin B (SR0155; Oxoid). One to three presumptive colonies (based on *Campylobacter*-specific colony morphology) were then selected from each tentatively positive plate, further sub-cultured on Horse Blood Agar (HBA), and incubated at 42°C for 48 h under two conditions (aerobic and microaerophilic). Presumptive *Campylobacter* isolates were selected by comparing colony characteristics on HBA in combination with that on modified CCDA. Confirmation was then conducted as per the AS using the Oxoid Biochemical Identification System (O.B.I.S.). Biochemically confirmed isolates of *Campylobacter* were then transferred to 5 mL of Mueller-Hinton (MH) broth (Oxoid) and incubated under microaerophilic conditions at 42°C for 48 h. A 1-mL aliquot was subsequently extracted from each culture and placed into cryo-protective tubes containing glycerol (final concentration of 20% [v/v]) before storing at −80°C.

### Cytotoxicity assay

The cytotoxicity assay was performed on HeLa cells according to a previously reported protocol (66). In brief, HeLa cells were grown in Dulbecco’s Modified Eagle’s Medium (DMEM) supplemented with 10% fetal bovine serum (FBS) and gentamicin (100 μg mL^−1^) and seeded on 96-well microtiter plates at a density of ~1.5×10^3^ HeLa cells (in 100 μL DMEM) per well. Cytotoxicity assays were performed prior to confluence at approximately 18 h post seeding. Ninety-five *Campylobacter* isolates from 79 different samples were tested in this assay. One pair of isolates was taken from 13 samples, while lone isolates were tested from the remaining 66 samples. *Campylobacter* strains were resuscitated from −80°C on MH agar incubated under micro-aerophilic conditions at 42°C for 48 h. Three colonies from each strain were inoculated into 3×3 mL MH broth for an overnight incubation under the same microaerophilic conditions. Each of the cultures were then transferred to Protein LoBind tubes (Eppendorf, Hamburg, Germany) before centrifugation at 4,000×*g* for 5 min followed by resuspension in 3 mL 1×PBS (pH 7.4). The suspension was pelleted again and resuspended in 3 mL 1×PBS (pH 7.4). One milliliter was then taken from this suspension to measure OD_600_ using plastic cuvettes. The remaining culture was sonicated in two 30-s bursts with 30-s rest intervals. This sonicate was then pelleted at 6,000×*g* for 5 min in order to remove unbroken cells. The resultant supernatant fractions were filtered with 0.22-μm pore-size syringe filters (Corning, NY, USA). One-milliliter aliquots from each sonicated suspension were stored in 1.5-mL tubes. Serial dilutions, 5× two-fold, were then conducted on sonicated preparations in DMEM. Diluted fractions were then applied to pre-prepared HeLa cells.

From serial dilutions, 2×100-μL aliquots were applied to duplicate tissue culture wells containing 18-h-old HeLa cells in 100 μL of DMEM. The plates were incubated at 37°C with 5% CO_2_ and examined daily for a period of 4 d for a distorted cell morphology using an inverted light microscope. Cytotoxin titers were expressed by dividing the highest dilution that caused a 50 to 75% distension of HeLa cells in a well that was observed by the OD_600_ for each of the test strains (66). *C. jejuni* NCTC 11168 was used as a positive control and *E. coli* K1 as a negative control.

### *In vitro* invasion and attachment assays for *Campylobacter*

*Campylobacter* isolates collected in the present study were resuscitated from frozen stock (as described earlier; Isolation of *Campylobacter*). The isolates were screened for their capacity to invade/attach to human epithelial cells of intestinal origin using the method outlined below.

### Inoculum preparation

Upon resuscitation, single colonies of *Campylobacter* (two for each test isolate) were transferred from MH agar plates to 5 mL of MH broth and incubated for 36 h under microaerophilic conditions (as described in Isolation of *Campylobacter*). Cultures were pelleted at 4000×*g* for 5 min and the supernatants were removed. Cell pellets were resuspended in 2 mL of 1×PBS (pH 7.4). Culture density was adjusted to an OD_600_ of 0.05 in order to maintain a concentration of approximately 3×10^8^ CFU mL^−1^. This suspension was then used to inoculate the invasion/attachment assay.

### Maintenance of Caco-2 monolayer cells

Caco-2 cells were routinely cultured in DMEM supplemented with 10% heat-inactivated fetal bovine serum (FBS), 1% non-essential amino acids, 1% (100×) glutamine, and 0.1% gentamicin (50.0 mg mL^−1^) in 75-cm^2^ flasks. Cells were grown to near confluence at 37°C in a humidified atmosphere (95% air and 5% CO_2_) before subculturing. In the invasion and attachment assays, separate sets of 24-well tissue culture plates were seeded with Caco-2 cells in pre-warmed 0.8 mL complete DMEM medium at a density of ~3.7×10^4^ cells mL^−1^. During the incubation, growth medium was changed every 2 or 3 d. Partially differentiated Caco-2 monolayers (~7 d old) were used with *Campylobacter* inocula for this assay. During the assay, pre-grown Caco-2 cells were washed three times with pre-warmed (at 37°C) DMEM, without FBS or gentamicin, to remove traces of the antibiotic and non-adherent bacteria. After the final wash, 1 mL of pre-warmed DMEM was added to each well, which was subsequently seeded with 40 μL of the prepared *Campylobacter* inoculum (~1.2×10^7^ cells well^−1^) in duplicate for each test isolate. Plates were incubated at 37°C in a humidified atmosphere (95% air and 5% CO^2^) for 4 h. Separate processing steps were subsequently performed for invasion and attachment assays on different days.

### Invasion and attachment assays

The invasion of Caco-2 cells by *Campylobacter* was performed in duplicate wells for each of the test strains following a previously reported method (23) with some modifications; a higher gentamicin concentration (450 μg mL^−1^) was applied to all assays to ensure the complete killing of extracellular bacteria (53) and the plates were incubated at 37°C for 1 h and 5% CO_2_. After the incubation, cells were washed three times with DMEM. Spent DMEM from the final wash was stored to assess the efficiency of killing bacteria outside Caco-2 cells. In attachment assays, the medium post-incubation was aspirated, and monolayers were rinsed five times with 1×PBS (pH 7.4) to remove non-adherent and loosely adhered bacteria. To liberate bacteria, washed cells were lysed with 1 mL 1% (v/v) Triton-X100 (Oxoid) in 1×PBS (pH 7.4) at room temperature for 5 min followed by gentle pipetting. Triton lysates (1 mL) from duplicate wells were subjected to 3×10-fold serial dilutions, with the last dilution being plated onto duplicate MH agar to count the number of attached *Campylobacter* (13, 25). Attachment assays were conducted in duplicate. *C. jejuni* NCTC 11168 was used as a positive control and *E. coli* K12 as a negative control for this assay. Invasion efficiency was calculated as the percentage of invaded bacteria to the total number of inoculated bacteria (56). The attachment efficiency of the test isolates was calculated as the percentage of attached bacteria to the total number of inoculated bacteria (22).

### Statistical analysis

All statistical analyses were performed using IBM SPSS Statistics Version 22. Binary logistic regressions were performed to assess the significance of relationships between *Campylobacter* detection and other water-quality and environmental parameters. Quantitative comparisons of HeLa cytotoxicity and Caco-2 attachment efficiencies among different spatial and temporal groups of *Campylobacter* isolates were tested using a one-way ANOVA. This was followed by post hoc tests (with Bonferroni adjustments) to assess the main effects and full factorial interactions among grouping variables (*P*<0.05 was considered to be significant). The Hazen function was used to calculate the 5^th^ and 95^th^ percentiles for non-normally distributed datasets (13).

## Results and Discussion

### Occurrence of *Campylobacter*

**Fig. 2:**
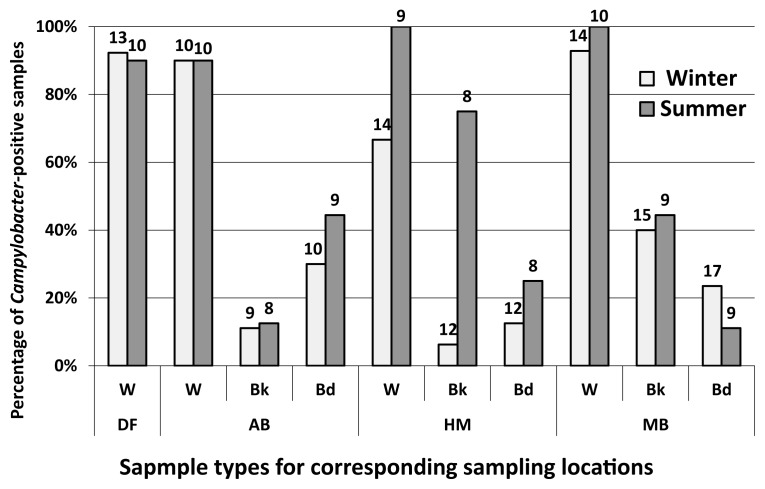
Incidence of *Campylobacter* in the Yarra River estuary (data labels represent the number of each sample type processed in this study). Sampling sites are on the X-axis (DF=Dights Falls, AB=Abbotsford, HM=Hawthorn, MB=Morell Bridge) and represent locations from a low to high salinity gradient, respectively. W=water samples, Bk=bank sediment samples, Bd=bed sediment samples

Among the 216 water and sediment samples tested during the two rounds of sampling, 117 yielded culturable *Campylobacter* (biochemically confirmed). These represented 62 and 55 *Campylobacter-*positive samples from winter and summer, respectively (Fig. 2). *Campylobacter* occurrence in the upstream freshwater site (DF) was 91% (*n*=23). This high incidence suggests that the upstream river (above DF) is a frequent source of culturable *Campylobacter* to the estuarine reaches of the Yarra River. This is consistent with previous findings on *E. coli* concentrations in the same system (19). The three estuarine sites in aggregate yielded a similar incidence to the freshwater reach (93% positive samples for culturable *Campylobacter*, *n*=67), indicating the likelihood of other sources of *Campylobacter* along the estuary.

The frequency of *Campylobacter*-positive samples did not significantly change along the downstream salinity gradient (*P*=0.97); 90% (*n*=20) in AB, 91% (*n*=23) in HN, and 96% (*n*=24) in MO. Since salinity has been reported as a stressor for *Campylobacter* (26, 36), the lack of a change along the salinity gradient indicates two possibilities—either *Campylobacter* from freshwater reaches had a high survival rate in the Yarra estuary or there were additional sources of *Campylobacter* compensating for salinity-induced inactivation. Whilst *Campylobacter* has been shown to survive for up to three weeks in the Yarra River estuary (70), the urbanized estuarine part of the Yarra River receives inputs from numerous stormwater drains that comprise a significant source of fecal organisms in the system (19). Furthermore, the Yarra River estuary attracts many wild birds that are common hosts of *Campylobacter* (1, 62).

Among all tested water samples (*n*=51), 90% were positive for *Campylobacter* during winter and 95% (*n*=39) during summer, with no significant temporal variation (*P*=0.39). To investigate the influence of atmospheric parameters on *Campylobacter* detection, relationships with daily maximum and minimum temperatures, solar radiation, relative humidity, and average daily rainfall were tested. The results of ANOVA and post hoc analyses demonstrated that none of these factors correlated with the occurrence of *Campylobacter*. However, correlations with environmental data may emerge with quantitative *Campylobacter* data.

Among estuarine sediment samples (*n*=61), 31% of bank sediment and 23% (*n*=65) of bed sediment samples were positive for *Campylobacter*. The detection of *Campylobacter* in bank sediments was 22% during winter (*n*=36) and 44% (*n*=29) during summer (*P*=0.07), while that in bed sediments was 21% (*n*=39) during winter and 27% (*n*=26) during summer (*P*=0.55). Regarding bank sediments, when the rates from individual sampling sites were compared, an increase in the frequency of detection was the greatest at the HN site; *i.e*. from 8% (*n*=12) during winter to 75% (*n*=8) during summer (Fig. 2). Similar results were also observed for water samples (86% [*n*=14] to 100% [*n*=9]) and bed sediments (8% [*n*=12] to 25% [*n*=8]) from winter and summer, respectively.

The occurrence rates observed within the sediments in the present study are generally at the higher end of the range reported from a limited number of similar studies conducted elsewhere; *i.e*. ranging between 0 and 24% (2–4, 34, 54), and demonstrate that bank and bed sediments are potential reservoirs of *Campylobacter* in urban estuaries. While it is generally assumed that *Campylobacter* in bank or bed sediments eventually die off, they have been reported to survive in these environments for as long as 21 d (70). Prior to dying off, *Campylobacter* may be resuspended to the overlying water column (under the influence of tidal fluctuations, water flow, or the movement of boats), which may influence the concentration of *Campylobacter* in the overlying water column and potentially add to the public health risks of recreational users.

### Cytotoxicity assay

**Fig. 3:**
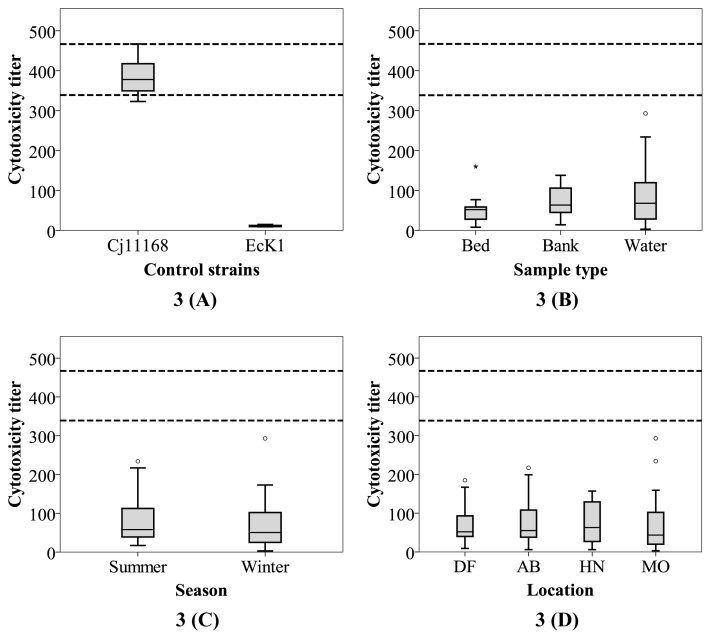
Cytotoxic potentials presented as toxin titers of test *Campylobacter* isolates and two control isolates; *C. jejuni* 11168 (a positive control and previously reported to have pathogenic potential) and *E. coli* K1 (a negative control) (A), grouped according to sample types (B), isolates grouped according to the season of sampling (C), and locations of sampling along the Yarra River (D). The two horizontal dashed lines represent the lower and upper boundaries of the 5^th^ and 95^th^ percentiles of the value for *C. jejuni* 11168. Abbreviations on the X-axis represent the following: DF=Dights Falls, AB=Abbotsford, HM=Hawthorn, MB=Morell Bridge

Ninety-one biochemically confirmed *Campylobacter* strains isolated from the three Yarra sites (50 during winter and 41 during summer) were tested for HeLa cell cytotoxicity. The toxin titers of these test isolates showed moderate variations when compared; 5^th^ and 95^th^ percentile values were 11 and 153, respectively. The *C. jejuni* NCTC 11168 control strain showed an average titer of 386 across 11 different batches of the experiment (5^th^ percentile value of 339 and 95^th^ percentile of 467; Fig. 3A). The 95^th^ percentile value of toxin titers for the estuarine strains was only 40% of the mean value for the control strain (which was originally a clinical isolate with known pathogenic potential). This result implies that the estuarine isolates showed significantly lower toxin titers than *C. jejuni* NCTC 11168. Similarly low titers have been observed among *Campylobacter* from non-clinical sources (including *C. jejuni* and *C. coli*), such as broiler chickens (10, 24).

Toxin production is widely accepted as an indicator of potential pathogenicity (28). However, previous studies primarily focused on investigating toxin production in a clinical setting. To the best of our knowledge, this is the first study to use the HeLa cell cytotoxicity assay to measure extracellular toxin titers of *Campylobacter* from surface water. Thus, difficulties are associated with finding similar studies for comparison because clinical studies often use strains with known high disease potential. Moreover, inter-study differences and uncertainties regarding multiple assay variables (*e.g*., cell lines used and assay conditions) may contribute to variations in toxin titers (17, 39). This is further complicated by the lack of uniformity in the manner in which cytotoxic potentials are reported in the literature; units including dilutions, titers, and percentages of cell death are all common (5, 45, 60, 66). Consequently, some uncertainty needs to be acknowledged in relation to the assumption that the estuarine strains in the present study with low cytotoxicity lacked the potential to be pathogenic to humans.

*Campylobacter* strains pathogenic to humans have been reported to display a wide range of toxin titers (from <40 to as high as >750) (5, 73). The present results suggest that while most of the *Campylobacter* isolated for the Yarra River estuary produce more active cytotoxins than *C. jejuni* NCTC 11168, which was previously described as pathogenic (5, 17), the environmental isolates investigated exhibited a lower capacity to produce cytotoxin. Notably, some strains lacking active cytotoxicity have been reported to have the capacity to invade or attach to human epithelial cells and have been implicated in human infection (5, 37). This is most likely due to toxin production representing only one of the multiple *Campylobacter* virulence factors. Thus, further investigations on a wider spectrum of virulence factors (gene complement and expression, whole genome sequencing, and *in vitro* and *in vivo* assays) within the isolates may provide a better understanding of their disease-causing potential.

A comparative analysis was performed to clarify whether HeLa cytotoxic activity is related to spatial, temporal, and isolation environment factors. Comparisons of strains isolated from the estuary to those from DF showed no significant variation in cytotoxicity (Fig. 3D). Similarly, spatial (water vs. bank vs. bed [Fig. 3B] and AB vs. HN vs. MO [Fig. 3D]) or temporal clusters (summer vs. winter; Fig. 3C) of isolates from within the estuary showed no significant variations (*P*>0.24 for all cases). Isolates from bed sediments had slightly lower cytotoxic potential than those from the other two sample types. While exposure to higher salinity in bed sediments may have influenced this result, further studies are required.

### Attachment and invasion capacity of environmental

**Fig. 4:**
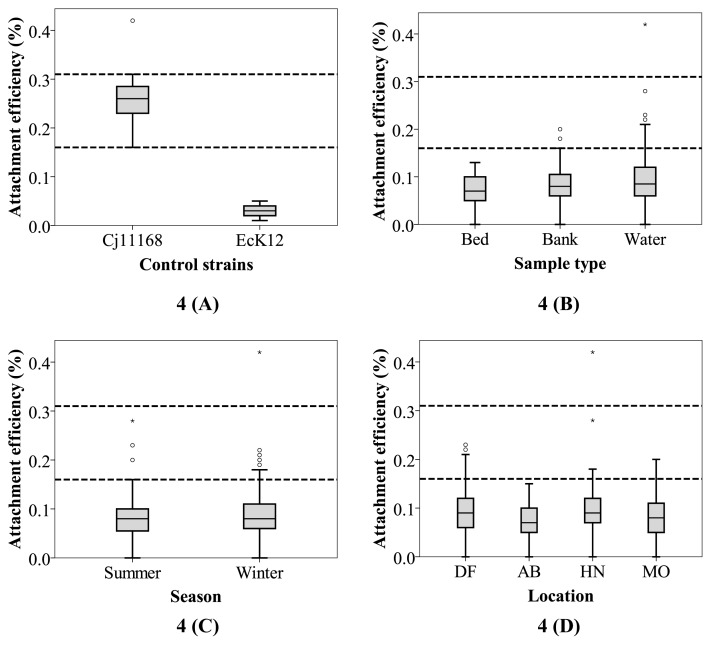
Attachment efficiency (percentage of bacterial cells attached to the Caco-2 monolayer to the total number of inoculated bacteria) after a 1-h incubation. The figures show attachment efficiencies for the two reference strains *C. jejuni* 11168 and *E. coli* K12 (A) and the test isolates grouped according to three sample types (B), two sampling seasons (C), and sampling locations (D). The two horizontal dashed lines represent the 5^th^ and 95^th^ percentile values for *C. jejuni* 11168. Abbreviations on the X-axis represent the following: DF=Dights Falls, AB=Abbotsford, HM=Hawthorn, MB=Morell Bridge.

Campylobacter The Caco-2 attachment capacities of the test isolates were generally low (95% CI 0.09–0.1%; Fig. 4). Regarding *C*. *jejuni* 11168, which is a highly virulent clinical strain (27), the mean attachment efficiency was 0.26% (5^th^ percentile of 0.16% and 95^th^ percentile of 0.31%; Fig. 4A) across 11 independent replicates. Similar attachment capacities of this reference strain (ranging between ~0.2 and 0.62%) have been reported previously (46, 68, 76).

The capacity to attach to the intestinal epithelium is commonly used to characterize *Campylobacter* of clinical origin for which a wide margin of Caco-2 attachment efficiency has been observed (ranging between 0.16% to as high as 5.4%) (14, 31, 43). Therefore, the 95^th^ percentile values for the biochemically confirmed *Campylobacter* isolates recovered from the Yarra River were lower (type result here) than the lowest value reported previously for clinical strains. As such, estuarine *Campylobacter* from the Yarra River had a relatively low level of Caco-2 attachment efficiency. However, among the isolates recovered from the Yarra River estuary, 6% (*n*=11; eight from winter and three from summer) had a higher attachment capacity than the 5^th^ percentile value observed for *C. jejuni* 11168, suggesting a potential public health risk. Two of these isolates were from bank sediments and nine from water samples. None of these isolates originated from bed sediments. Among water samples, four were from DF, three from HN, and four from MO. While these isolates were collected on six different sampling days, five (two from DF, two from MO, and one from HN) came from four different samples collected on a single day, suggesting that a consistent source of these high-attachment *Campylobacter* was present in this system on that particular day. It was not possible to establish a clear correlation with any of the environmental parameters recorded by the BOM, Victoria, which may partly be due to the low number of data points. However, it was noted that, among these environmental parameters, the average daily rainfall on the sampling day or the day before was 0.2 mm or higher on four out of the six sampling days (data not shown) on which these high-attachment *Campylobacter* were found. This result suggests that further investigations on the relationship between rainfall and public health risks are warranted (52).

The role of spatial and/or temporal factors in attachment capacity was investigated. The mean attachment efficiency values for the four sampling locations (Fig. 4D) were found to be 0.10% (95^th^ CI 0.09–0.12%) for DF, 0.08% (95^th^ CI 0.07–0.09%) for AB, 0.11% (95^th^ CI 0.09–0.13%) for HN, and 0.09% (95^th^ CI 0.08–0.10%) for MO. The difference among the *Campylobacter* isolated from different sampling locations was not significant (*P*=0.23). Similarly, attachment efficiencies for the isolates from three different sample types (Fig. 4B), namely, water (mean 0.10%, 95^th^ CI 0.09–0.11%); bank sediment (mean 0.10%, 95^th^ CI 0.08–0.11%); and bed sediment (mean 0.08%, 95^th^ CI 0.06–0.11%) did not show any significant spatial difference (*P*=0.56). Furthermore, despite the occurrence of *Campylobacter* in bank sediments being more frequent during summer (Fig. 2), no significant difference (*P*=0.19) was observed in the attachment efficiency of isolates between summer (mean 0.09%, 95^th^ CI 0.08–0.10%) and winter (mean 0.10%, 95^th^ CI 0.09–0.11%; Fig. 4D).

While estuarine water quality and environmental parameters did not significantly affect the spatial or temporal pattern of *Campylobacter* in the estuary, it is important to note that the 95^th^ CI values for *Campylobacter* from bed sediments (as opposed to those from water or bank sediments) were lower than that of the pathogenic control (Fig. 4B). Fecal organisms that belong to the estuarine bed sediment withstand exposure to higher levels of salinity in general and this is particularly the case for a microtidal estuary, such as the lower Yarra River (42). While high salinity (generally greater than 1.7% [w/v]) is known to induce a stress response in *Campylobacter* (36, 47), the effects of this stressor on its virulence properties (including epithelial attachment) are unknown and worthy of further investigation. Based on other confounding factors (*e.g*., the lack of sunlight exposure, water temperature, and inherent genetic variations) that may also influence the interplay, the lower virulence of *Campylobacter* in the bed sediment may be important for public health outcomes. If confirmed, this may be an important consideration for conducting QMRAs in estuarine systems.

The invasion of the intestinal lining represents a severe form of infection and a critical virulence factor (9, 48, 57). Previous studies reported a correlation between *Campylobacter* invasiveness, observed in Caco-2, and clinical manifestation (48, 59). Therefore, invasion assays were conducted to further characterize estuarine isolates. All isolates were screened for their invasiveness in Caco-2 cells because this cell line has been proposed as a model for enteric invasion (23). Culturable *Campylobacter* counts from Caco-2 lysates for individual isolates were compared to the background counts of the final wash solution obtained immediately before the Caco-2 monolayers were lysed (data not shown). Background counts were similar to the counts from lysates. Hence, we concluded that the isolates were either non- or very weakly invasive. Similar weak invasiveness (less than 0.01%) has been commonly reported in the literature for non-*jejuni Campylobacter* spp. (43, 55).

### Defining the pathogenic potential of *Campylobacter* isolates

The present results suggest that none of the biochemically confirmed *Campylobacter* isolated from the Yarra River estuary had cytotoxic titers greater than the 5^th^ percentile value observed for *C. jejuni* 11168 (Fig. 3). At the same time, only 3.7% of these strains had a Caco-2 attachment capacity that was greater than the 5^th^ percentile of the same control strain (Fig. 4). No strains displayed invasive capabilities. A correlation was not observed (*P*=0.5) between the cytotoxicity titers and attachment efficiencies of these isolates. Furthermore, isolates with the top 10% cytotoxin titer were compared to those with the top 10% attachment efficiency (data not shown); only one isolate (isolated from the water of HN during summer) belonged to both groups, while the remainder did not match. This apparent lack of an overlap between the high cytotoxicity and high attachment groups suggests that the attachment and cytotoxicity capacities of *Campylobacter* isolated from the Yarra River estuary are independent. This hypothesis is supported by previous findings of cytotoxin-negative strains being isolated from the blood and feces of infected patients and having the capacity to attach to the epithelial cells of human origin (5, 37). This ultimately indicates that strains that do not appear to possess pathogenic potential using one assay may not lead to similar conclusions when tested by other assays. As such, the evidence provided here via our attachment, invasion, and cytotoxicity assays suggests that the assumption that all environmental strains are 100% virulent, which is common in QMRAs (29, 75, 77), is not sound when all virulence determinants are considered.

## Conclusion

The present study investigated spatial and temporal variations in *Campylobacter* contamination and their pathogenic potential in an urban estuary. The results obtained indicate that the water column and sediments found within the estuary are frequently contaminated with biochemically confirmed culturable *Campylobacter*, although overall Caco-2 attachment efficiency was low (only 6% greater than the 5^th^ percentile of the positive control). Furthermore, the results for HeLa cytotoxicity suggest overall low cytotoxin titers (none greater than the 5^th^ percentile of the control strain) and none of the estuarine isolates invaded the Caco-2 cell line. When considered holistically, the isolates from the Yarra River estuary appeared to have overall low pathogenic potential, at least at the phenotypic level. In consideration of multiple virulence factors and the use of different methods (attachment, invasion and cytotoxic assays) to assess pathogenic potential, as used in the present study, the assumption that all environmental strains are 100% virulent, which is common in QMRAs, may not be appropriate.

The present study used multiple *in vitro* assays to investigate potential pathogens in order to understand the risks to human health in an urban estuarine context. The consistency between the values observed in this study for the experimental control strain and corresponding literature values (including other similar pathogenic strains) implies that the *in vitro* assays used in the present study offer valuable means of virulence characterization for individual isolates. The results presented herein may provide significant insights into human health risks from *Campylobacter* in urban estuaries.
